# Simultaneous Antagonism at H3R/D2R/D3R Reduces Autism-like Self-Grooming and Aggressive Behaviors by Mitigating MAPK Activation in Mice

**DOI:** 10.3390/ijms24010526

**Published:** 2022-12-28

**Authors:** Nermin Eissa, Mohamed Al Awad, Shilu Deepa Thomas, Karthikkumar Venkatachalam, Petrilla Jayaprakash, Sicheng Zhong, Holger Stark, Bassem Sadek

**Affiliations:** 1 Department of Pharmacology & Therapeutics, College of Medicine and Health Sciences, United Arab Emirates University, Al Ain P.O. Box 15551, United Arab Emirates; 2 Zayed Bin Sultan Center for Health Sciences, United Arab Emirates University, Al Ain P.O. Box 15551, United Arab Emirates; 3 Department of Biomedical Sciences, College of Health Sciences, Abu Dhabi University, Abu Dhabi P.O. Box 59911, United Arab Emirates; 4 Institute of Pharmaceutical and Medicinal Chemistry, Heinrich Heine University Düsseldorf, Universitaetsstr. 1, 40225 Düsseldorf, Germany

**Keywords:** histamine H3 receptor antagonist, dopamine D2/D3R antagonist, autistic spectrum disorder, repetitive self-grooming, aggressive behaviors, MAPK proteins, neuroinflammation, histamine, dopamine, BTBR mice

## Abstract

Dysregulation in brain neurotransmitters underlies several neuropsychiatric disorders, e.g., autism spectrum disorder (ASD). Also, abnormalities in the extracellular-signal-regulated kinase (ERK)/mitogen-activated protein kinase (MAPK) pathway pave the way for neuroinflammation, neurodegeneration, and altered learning phenotype in ASD. Therefore, the effects of chronic systemic administration of the multiple-targeting antagonist ST-713 at the histamine H3 receptor (H3R) and dopamine D2/D3 receptors (D2/D3R) on repetitive self-grooming, aggressive behaviors, and abnormalities in the MAPK pathway in BTBR T + Itpr3tf/J (BTBR) mice were assessed. The results showed that ST-713 (2.5, 5, and 10 mg/kg, i.p.) mitigated repetitive self-grooming and aggression in BTBR mice (all *p* < 0.05), and the ameliorative effects of the most promising dose of ST-713 (5 mg/kg, i.p.) on behaviors were completely abrogated by co-administration of the H3R agonist (*R*)-α-methylhistamine or the anticholinergic drug scopolamine. Moreover, the elevated levels of several MAPK pathway proteins and induced proinflammatory markers such as tumor necrosis factor (TNF-α), interleukin-1β (IL-1β), and IL-6 were significantly suppressed following chronic administration of ST-713 (5 mg/kg, i.p.) (all *p* < 0.01). Furthermore, ST-713 significantly increased the levels of histamine and dopamine in hippocampal tissue of treated BTBR mice (all *p* < 0.01). The current observations signify the potential role of such multiple-targeting compounds, e.g., ST-713, in multifactorial neurodevelopmental disorders such as ASD.

## 1. Introduction

Autism spectrum disorder (ASD) is a multifactorial developmental disorder of neurological origin and is identified by deficiencies in multiple traits, including difficulties in social communication and interaction, mental inflexibility, and repetitive manners accompanied by aggressive behavior [1,2,3,4,5,6]. The domain of repetitive behavior includes uncontrolled motoric stereotyping, a rigid insistence on uniformity, and a reluctance to change [7,8]. The mitogen-activated protein kinase (MAPK) signaling pathway is an intracellular signaling pathway that controls a variety of intracellular processes, and several genes involved in ASD have been found to interact with this MAPK signaling pathway [9,10,11]. In addition, several studies reported that ASD-related endophenotypes can be caused by dysregulation of the extracellular-signal-regulated kinase (ERK) signaling pathway, which correlates with the pathophysiology observed in 16p11.2del animals [12]. Moreover, MAPKs are a group of protein kinases that are activated by phosphorylation of tyrosine and threonine residues. In previous preclinical studies, ERK family, particularly the p38 kinase family and the c-JUN N-terminal kinase family (JNKs), was reported to induce cytokine production, particularly production of tumor necrosis factor-α (TNF-α). Also, JNK has been described to be involved in the TNF-α-mediated neuroinflammation and neurodegeneration [13,14,15,16]. Interestingly, recent research observations have shown that pharmacological inhibition of ERK signaling was able to reverse the increased dendritic arborization found in two animal models of ASD [15,16]. In addition to the MAPK signaling pathway, MAPK is also thought to be responsible for the activation of microglia, which has been found to induce chronic neuroinflammation leading to neuronal death mediated by neuronal apoptosis, necrosis and production of TNF-α [17]. As a result, the chronic neuroinflammatory condition is often associated with cognitive and behavioral abnormalities that resemble the ASD phenotype. Many studies have reported that immune system complications are common in ASD and are accompanied by increased activation of brain glial cells and increased levels of interferon, interleukin (IL)-1β, IL-6, IL-12, and tumor necrosis factor (TNF)-α [18,19]. It has also been reported that long-term neuronal inflammation is associated with reduced social behavior, which is comparable to the response of children exposed prenatally to VPA [20]. Furthermore, several previous studies have suggested the role of certain brain neurotransmitters in the early stages of brain development and are associated with the onset and development of ASD [1]. It is widely accepted that the neurotransmitters acetylcholine (ACh), dopamine (DA), and histamine (HA) are critical to cognitive function of numerous neuropsychiatric disorders, including ASD [21,22]. Therefore, novel multiple-active agents are emerging and studying the development of new inventive drugs is an interesting field of research [4,23,24,25,26,27,28,29,30,31]. HA has been shown to influence behavior in disorders that overlap with ASD, such as Alzheimer’s disease (AD), schizophrenia (SCH), anxiety, Tourette’s syndrome (TS), and narcolepsy. Antagonism of histamine receptors (H1-3Rs) appears to reduce symptoms and some behaviors in individuals with ASD and related animal models, according to preliminary clinical and preclinical studies [32]. In the brain, the tuberomammillary nucleus (TMN) has been found to show a high expression of histidine decarboxylase (HDC), an enzyme which is essential for the biosynthesis of brain histamine. Consequently, the histaminergic system (HS) is associated with the outcome of this syndrome through the premature termination codon (W317X) in the HDC gene seen in TS. Several population-based studies have found an important link between TS and ASD [33], raising the possibility that brain HS is dysregulated in ASD. The histamine H3 receptor (H3R) was discovered in 1983 as a presynaptic autoreceptor and found to adversely affect the production and release of HA as well as other neurotransmitters such as DA and ACh in various brain regions [34,35]. Selective histamine H3 receptor (H3R) antagonists/inverse agonists have the ability to improve the cognitive behavior observed in SCH and ASD [28,31,36,37]. Regarding the DA system, previous genetic research has revealed an association between ASD and mutations in the dopaminergic system [24,38,39,40,41,42], signifying the influence of dopaminergic system dysfunction in ASD-associated cognitive deficits [43,44]. Several of the behavioral characteristics associated with ASD have been found to correlate with deficiencies in striatal DA neurotransmission [45]. Previous research has also shown that aberrant DA signaling can cause or aggravate the ASD symptoms [46,47], demonstrating the importance of studying brain dopaminergic dysfunction. In addition, chemical and histochemical tests in the brains of individuals with ASD have shown the loss of nicotinic receptors [48]. Postmortem examinations also showed a significant decline in the mRNA levels of the α-7 receptor [49]. Another interesting study showed that the expression of the α-4 nAChR subunit was down-regulated in the frontal cortex and up-regulated in the cerebellum [50]. ASD and other cognitive deficits in neuropsychiatric disorders also appear to improve with brain ACh-enhancing approaches.

Given that dysregulation of brain HA, DA, and ACh is associated with repetitive and aggressive behavior in ASD while effective potent pharmacological treatments are sought, this study examines the mitigating effects of chronic administration of a new test compound with antagonist affinities to H3Rs and D2Rs/D3Rs, namely ST-713 [3-(2-chloro-10*H*-phenothiazin-10-yl)-*N*-methyl-*N*-(4-(3-(piperidin-1-yl)propoxy)benzyl)-propan-1-amine], on ASD-like repetitive self-grooming and aggressive behaviors in male BTBR mice by applying a battery of behavioral tasks. The multiple H3R/D2R/D3R antagonist ST-713 showed a potent antagonist affinity and high selectivity profile to histamine *h*H3Rs (*K*_i_ = 1.21 nM), and balanced high and selective affinities to dopaminergic receptor subtypes *h*D2Rs (*K*_i_ = 41 nM) and *h*D3Rs (*K*_i_ = 50 nM) [31]. In addition, the effect of ST-713 on the levels of proinflammatory markers in the brain, as well as the neurotransmitters DA and HA in the hippocampus and cerebellum, in treated BTBR mice was examined. To analyze our observations, we evaluated the potential of CNS-penetranting H3R agonist (*R*)-α-methylhistamine, the H1R antagonist pyrilamine, the H2R antagonist zolantidine, and the cholinergic antimuscarinic scopolamine to reduce the ST-713-provided effects, to interpret the possible involvement of hippocampal and/or cerebellar DA and HA in the response generated by the multiple-targeting test compound ST-713. 

## 2. Results

The animals used in this study received all test compounds following chronic treatment regimens and for a duration of 21 days. The control B6 mice (group 1) were administered VEH. BTBR mice with ASD-like behaviors were treated with VEH (group 2) and served as the control group of mice with autistic-like features. A range of different doses (2.5–10 mg/kg, i.p.) were administered to BTBR mice (groups 3–5). The reference drug Chlorpromazine (1.5 mg/kg) was administered to BTBR mice (group 6). For the further abrogative studies, 5 mg/kg of the multiple-active test compound was co-administered with pyrilamine (10 mg/kg, group 7), zolantidine (10 mg/kg, group 8), scopolamine (0.3 mg/kg, group 9), or (*R*)-α-methylhistamine (10 mg/kg, group 10) in BTBR mice. To exclude the confounding effects of any possible H3R-agonism, BTBR mice were injected with (*R*)-α-methylhistamine (10 mg/kg, group 11) alone. In addition to the 11 groups described above, four groups of six B6 mice each received ST-713 (2.5–10 mg/kg) as well as the reference medication chlorpromazine to exclude any confounding properties of test compounds on behaviors of control B6 mice. For the self-grooming assessment, a total of 15 mouse groups (six mice/group) were used. For the biochemical measurements such as pro-inflammatory cytokine estimations (TNF-α, IL-1β, and IL-6), three mice/group were used. For biochemical estimation of histamine and dopamine brain levels, three mice/group were utilized. Moreover, to obtain results for Western blot analysis and Iba-immunofluorescence staining, three mice/group were used.

### 2.1. ST-713 Reduced Recurrent Self-Grooming in Autistic Mice without Any Effect on Anxiety-Like Behavior or Locomotion in an Elevated Platform Test

**Figure 1:**
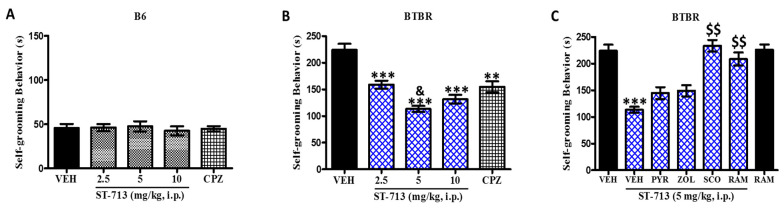
ST-713 reduced repetitive self-grooming behaviors in BTBR mice. The total duration of the self-grooming experiment was 10 min. No significant difference was found in the amount of time spent in self-grooming for B6 mice irrespective of their chronic systemic treatments (21 days) with VEH, ST-713 (2.5–10 mg/kg), or chlorpromazine (CPZ, 1.5 mg/kg) (**A**). BTBR mice treated with the multiple-active test compound at varying dosages, or reference medication chlorpromazine (**B**). The results observed for abrogative properties of pyrilamine (PYR, 10 mg/kg), zolantidine (ZOL, 10 mg/kg), scopolamine (SCO, 0.3 mg/kg), or (*R*)-α-methylhistamine (RAM, 10 mg/kg) on the enhancements obtained with ST-713 on repetitive self-grooming behaviors of mice with ASD-like behaviors (**C**). Figures show mean ± SEM (n = 6). ** *p* < 0.01 vs. VEH-receiving BTBR group. *** *p* < 0.001 vs. VEH-treated BTBR group. ^&^ *p* < 0.01 vs. chlorpromazine-treated BTBR group. ^$$^ *p* < 0.01 vs. ST-713-(5 mg)-receiving BTBR group.

The repetitive self-grooming behaviors of BTBR mice treated with vehicle, ST-713 doses, or the standard drug chlorpromazine are depicted in Figure 1A–C. A two-way ANOVA was performed to evaluate the strain (BTBR and B6), treatment (different doses, standard drug, and H3R agonist), and strain × treatment interaction. As expected, a significant impact was present with statistics of [*F*_(1,50)_ = 447.90, *p* < 0.001], [*F*_(4,50)_ = 16.78, *p* < 0.001], and (*F*_(4,50)_ = 16.38, *p* < 0.001], respectively. In this experiment, post hoc analysis revealed that VEH-treated BTBR mice spent considerably longer cumulative self-grooming time (224.33 ± 10.13 s) than B6 control mice (49.34 ± 5.25 s) (*p* < 0.001). However, self-grooming behavior time was significantly reduced following chronic administration of ST-713 at 2.5 mg/kg (*p* < 0.001), 5 mg/kg (*p* < 0.001), and 10 mg/kg (*p* < 0.001) or the standard drug chlorpromazine (*p* < 0.01) (Figure 1B). There was no statistical significance observed between the effects of ST-713 doses of 2.5 (*p* = 0.75) and 10 mg/kg (*p* = 0.48) and the standard medication chlorpromazine on self-grooming behavior of BTBR mice. However, the modulating effects of systemic administration of ST-713 at 5mg/kg (*p* < 0.01) on self-grooming behaviors were substantially greater than those of chlorpromazine. On the other hand, systemic co-injection of scopolamine and the H3R agonist (*R*)-α-methylhistamine increased self-grooming time (all *p* values <0.01) compared to ST-713-administered BTBR mice (Figure 1C). Contrarily, systemic co-administration with the centrally acting histamine H1R antagonist pyrilamine or the histamine H2R antagonist zolantidine was not able to change the improved effects observed with the administration of a 5 mg/kg dose of ST-713 alone on self-grooming behaviors, with [*p* = 0.60] and [*p* = 0.78], respectively (Figure 1C). Importantly, chronic systemic pretreatment with VEH, ST-713, or chlorpromazine did not modify the self-grooming time spent by tested control B6 mice (Figure 1A). Additionally, and in comparison, to VEH-treated BTBR mice, time spent in self-grooming by BTBR mice was not affected following chronic systemic treatment with (*R*)-α-methylhistamine (10 mg/kg, i.p.) (Figure 1C). 

### 2.2. ST-713 Mitigated Aggressive Behaviors of BTBR Mice

**Figure 2:**
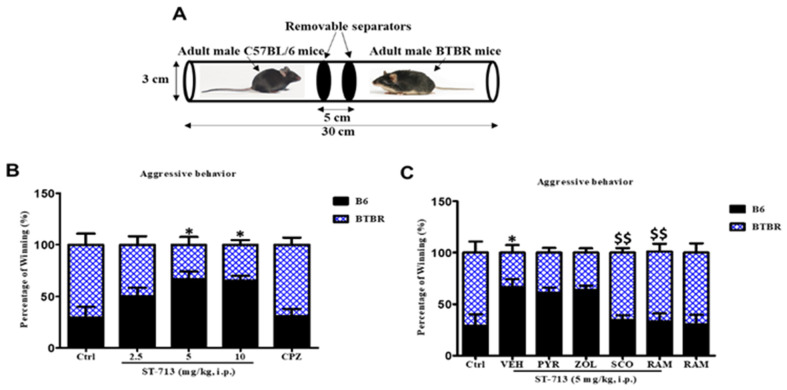
ST-713 reduced aggressive behavior of BTBR mice in a behavioral tube test. Experimental setup for the conducted test tube to assess aggression (**A**). The results obtained were percentage winning (**B**), and the results observed for abrogative properties of pyrilamine, zolantidine, scopolamine, or (*R*)-α-methylhistamine on the enhancements obtained with ST-713 on aggressive behaviors of autistic-like mice (**C**). Figures show mean ± SEM (n = 6). * *p* < 0.05 vs. VEH-treated BTBR group. ^$$^ *p* < 0.01 vs. ST-713-(5 mg)-treated BTBR group.

The social dominance tube test was used to evaluate the social approach–avoidance behaviors of tested animals. To avoid mice injury, real fights between mice were prevented by this approach. Based on this experiment, BTBR mice showed more aggression than control B6 mice (Figure 2A). In fact, VEH-treated BTBR mice won 70.83 ± 10.89% [*F*_(1,10)_ = 6.10; *p* < 0.05] of the matches against control B6 mice. However, systemic chronic pretreatment with ST-713 (5 and 10 mg/kg, i.p.) mitigated the BTBR mice’s aggressive behavior with 33.33 ± 7.61% and 34.67 ± 4.57% of won matches, respectively, (all *p* < 0.05) (Figure 2B). Contrarily, the lowest dose of 2.5 mg/kg of test compound and chlorpromazine did not show significant effects on the reduction in aggressive behavior of pretreated BTBR mice, with *p* = 0.21 and *p* = 0.91, respectively (Figure 2B). Furthermore, statistical analyses of the obtained results showed that the given response of ST-713 on the reduction in aggression of BTBR mice was abrogated following chronic systemic co-administration of scopolamine or (*R*)-α-methylhistamine, with [*F*_(1,10)_ = 10.83; *p* < 0.01] and [*F*_(1,10)_ = 8.00; *p* < 0.01], respectively (Figure 2C). However, co-administered CNS-penetrant antagonists for histamine H1- or H2Rs failed to revoke the mitigating effects of the test compound on aggressive behaviors of autistic animals (all *p* values > 0.05) (Figure 2C).

### 2.3. ST-713 Modulating Effects on MAPK Pathway Protein in B6 and BTBR Autistic Model

**Figure 3:**
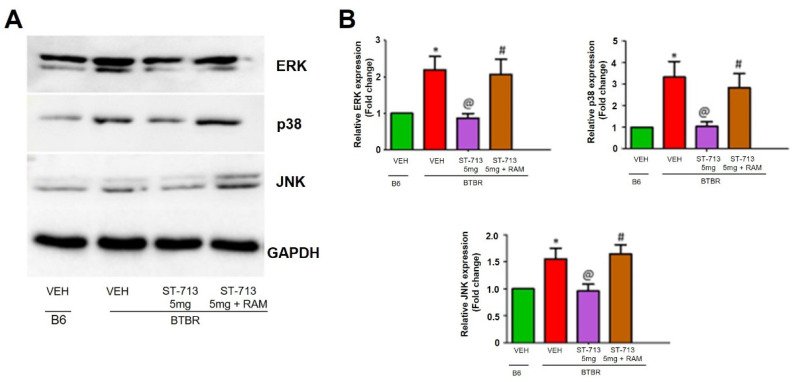
ST-713 modulated MAPK pathway proteins in B6 and BTBR mice. (**A**) Western blot patterns of anti-ERK, anti-p38, and anti-JNK levels in the brain tissues of all tested mice. The protein expression levels and corresponding results are depicted as fold change to the controls. The BTBR mice showed significantly elevated levels of ERK, p38, and JNK when compared to the VEH-treated B6 group. BTBR mice pretreated with ST-713 (5 mg/kg, chronic administration) significantly decreased the expression levels of ERK, p38, and JNK. (**B**) Bar graph represents the relative intensities of ERK, p38, and JNK levels. The multiple-active test compound at a dose of 5 mg/kg provided beneficial effects which were abrogated by (*R*)-α-methylhistamine (RAM, 10 mg/kg). The column heights represent the mean ± SEM (n = 3). ^@^ *p* < 0.05 vs VEH-treated BTBR group: * *p* < 0.05 vs. VEH-treated B6group; ^#^ *p* < 0.05 vs. ST-713 (5 mg/kg).

The effect of chronic injections of the multiple-active test compound ST-713 at 5 mg/kg on the expression of MAPK pathway proteins in BTBR are indicated in Figure 3A,B. The expression of ERK, p38, and JNK was detected as bands with molecular weights of approximately 42 kDa, 38 kDa, and 48 kDa, respectively (Figure 3A). The mean protein expression in the control samples was designated as fold change 1 in the graph. Densitometric analysis of saline-exposed BTBR mouse brain hippocampal tissue revealed significantly (*p* < 0.05) elevated expression of ERK, p38, and JNK proteins compared with the VEH-treated B6 control mice. However, and in the hippocampus, chronic systemic treatment with ST-713 significantly reduced the expression of ERK, p38, and JNK proteins in ST-713-treated treated BTBR mice, and these reducing effects on protein expression were reversed following systemic chronic co-injection with the CNS-penetrating H3R antagonist (*R*)-α-methylhistamine (Figure 3B). 

### 2.4. ST-713 Reduces Brain Inflammation Visualized by Anti-Ionized Calcium Binding Adaptor Molecule-1 (Iba-1) Immunofluorescence Staining

**Figure 4:**
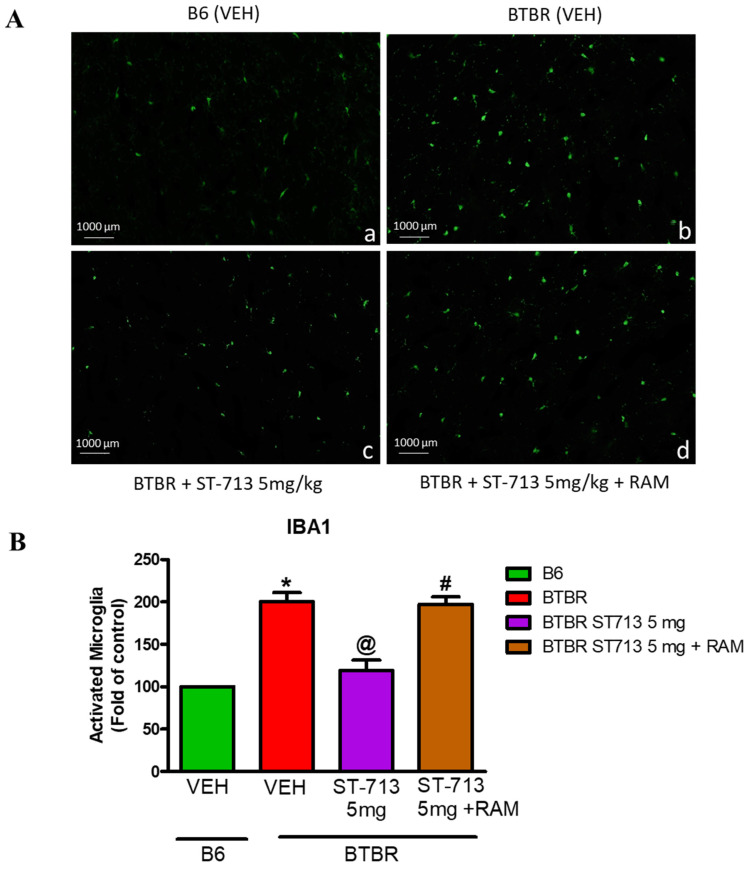
ST-713 inhibited microglia activation in BTBR mice. Immunofluorescence staining of iba-1-positive microglial cells in the brain sections of experimental B6 and BTBR mice. (**A**) (a,b) B6, BTBR VEH-treated mice (c) ST-713 (5 mg/kg) administered to autistic mice (d) ST-713 (5 mg/kg) co-administered with RAM (10 mg/kg) to BTBR mice. The densitometric evaluation for activated microglia was performed with ImageJ. (**B**) VEH-treated BTBR mouse brain shows significantly increased levels of iba-1-positive activated microglial cells compared to VEH-treated B6 mice. Chronic systemic pretreatment with ST-713 (5 mg/kg) in BTBR mice significantly reduced the level of iba-1-positive activated microglial cells compared to VEH-treated BTBR mice. Chronic co-injection with RAM (10 mg/kg) abolished the beneficial outcomes provided by a 5 mg/kg dose of ST-713. The bar diagram represents percentage mean of three samples ± SEM (n = 3); * (*p* < 0.05) vs. VEH-treated B6; ^@^ *p* < 0.05 vs. VEH-treated BTBR group; ^#^ *p* <0.05 vs. ST-713 (5 mg/kg).

As described earlier, microglial activation in brain segments of the cerebellum was carried out using immunofluorescence staining to analyze the levels of iba-1-positive microglial cells [3,51]. The iba-1 quantification of VEH-treated BTBR mice suggested a significant increase in expression compared to B6 control mice (*p* < 0.05) (Figure 4A,B). However, ST-713 (5 mg/kg) reduced microglial activation, evidenced by reduced iba-1-stained cells (*p* < 0.05) (Figure 4A,B). In addition, systemic co-administration with the brain-penetrating H3R agonist (*R*)-α-methylhistamine completely reversed the effect obtained in murine autistic model mice with ST-713 alone (*p* < 0.05) (Figure 4A,B). 

### 2.5. ST-713 Pretreatment Modulates Proinflammatory Cytokine Levels in the BTBR Brain

The proinflammatory cytokines IL-1β, IL-6, and TNF-α were evaluated in the ST-713-pretreated BTBR mice hippocampal and cerebellar tissues. (Table 1). A remarkable elevation in all three proinflammatory cytokines in BTBR mice brains was observed compared to B6 mice brains (Table 1) (all *p* values < 0.05). The up-regulation of inflammatory responses may be a reason behind this increment, which was subsidized following systemic administration of ST-713 in three doses (2.5, 5, or 10 mg/kg) or chlorpromazine in BTBR mice (all *p* < 0.01) (Table 1). Additionally, and following co-administration with (*R*)-α-methylhistamine, the reducing effects of the test compound on hippocampal and cerebellar levels of TNF-α, IL-1β, and IL-6 were reversed (all *p* values < 0.05). 

### 2.6. ST-713 Pretreatment Improves HA and DA Levels in the Brains of BTBR Mice

Our observed results indicated a significant reduction in the hippocampal and cerebellar levels of HA and DA in the VEH-treated BTBR mice when compared to VEH-treated control B6 mice (all *p* values < 0.05) (Table 2). However, the multiple-active test compound ST-713 was able to reduce the increased levels of HA and DA in hippocampal tissues of treated BTBR mice, with [*F*_(1,10)_ = 36.08; *p* < 0.001] and [*F*_(1,10)_ = 7.48; *p* < 0.05], respectively, and as compared with VEH-treated BTBR mice (Table 2). Also, the average of the cerebellar levels of HA was significantly mitigated following treatment with ST-713 [*F*_(1,10)_ = 21.31; *p* < 0.001]. Contrarily, ST-713 failed to alter the levels of DA in the cerebellum of the treated BTBR group [*F*_(1,10)_ = 0.09; *p* = 0.76] (Table 2). Similarly, chlorpromazine had no effect on the levels of HA and DA in either of the two brain regions assessed in BTBR mice (Table 2). Additionally, upon systemic co-administration of H3R agonist (*R*)-α-methylhistamine, statistical analyses of observed results showed that the test-compound-provided modulating effects on brain HA and DA were reversed in assessed mice with ASD-like features (all *p* < 0.05) (Table 2). 

## 3. Discussion

Changes in brain histaminergic, dopaminergic, and cholinergic neurotransmissions in the brain are thought to affect the phenotypic aspects of ASD-related features [2,23,32,44,52,53]. Therefore, the aim of this study was to investigate whether chronic systemic injection of ST-713 had a mitigating effect on ASD-like repetitive self-grooming and aggressive tendencies in BTBR mice. ST-713 and the reference drug chlorpromazine successfully reduced the pronounced repetitive self-grooming behaviors in BTBR mice. The provided effects of ST-713 were comparable to those of the reference drug chlorpromazine, with ST-713 (5 mg/kg) being the dose shown to be most effective. In other abrogative studies, the improvement in repetitive self-grooming behavior provided by ST-713 was completely abrogated by the antimuscarinic compound scopolamine or the brain-penetrating H3R agonist RAM. These latter results are supported by our theory that ST-713 has beneficial effects on repetitive self-grooming behaviors through modulation of various released neurotransmitters, namely via the H3-autoreceptor and H3-heteroreceptor antagonism, including HA and ACh, respectively. Furthermore, these results from the current series of experiments are consistent with previous observations by our group and others [32,52,53]. In contrast, to the reversal effects observed with concomitant administration with the centrally acting H3R agonist (*R*)-α-methylhistamine or the antimuscarinic drug scopolamine, CNS-penetrant H1- or H2R antagonist did not counteract the effects obtained for our test compound ST-713 on self-grooming behaviors. The latter observations supported our hypothesis that interactions with H3Rs alone was responsible for these noticeable behavioral improvements. Inspired by previous prominent studies of the underlying mechanisms contributing to social dominance/aggression in mice by Wang et al., 2011 and Saxena et al., 2018 [54,55], the tube test was applied to evaluate the social aggression as comparatively tractable indices of social interactions in rodents. The observed results showed that ST-713 mitigates the aggression of BTBR mice, and as observed in self-grooming behaviors the ST-713 (5 mg/kg)-provided reductions in aggressive behaviors were reversed following systemic co-administration with the antimuscarinic scopolamine or (*R*)-α-methylhistamine. The latter findings corroborated our present findings for ST-713 on self-grooming behaviors, indicating that HA and ACh were also involved in the behavioral effects of the multiple-active test compound. The inability of the reference drug chlorpromazine to alleviate social-like aggressive behaviors in BTBR mice in the test tube assessment indicates that modulation of brain dopaminergic neurotransmission does not significantly contribute to the level of aggression of the BTBR mice tested. In another set of experiments, it was found that HA and DA levels were significantly reduced in the hippocampal and cerebellar regions of autistic mice compared to B6 controls. However, chronic systemic administration of 5 mg/kg of our multiple-active test compound ST-713 significantly modulated brain HA and DA levels in the hippocampus and cerebellum of treated BTBR mice. Furthermore, systemic co-administration of the H3R agonist (*R*)-α-methylhistamine counteracted the effects of our test compound on DA and HA in the brain of tested BTBR mice. The latter results are crucial and demonstrate the importance of H3Rs and their involvement in the promising ST-713-mediated effects. Also, the effects shed light on the multiple-active properties of ST-713 influencing brain neurotransmitters, namely ACh and DA concurrently, which appears to play a crucial role in the pathophysiology of ASD-like features of BTBR mice. Furthermore, the observed mitigating effects of ST-713 (5 mg/kg) in BTBR mice were not reversed when mice were pretreated with the centrally-acting H1R antagonist pyrilamine or H2R antagonist zolantidine, a finding which similarly suggests that histaminergic pathways through activation of postsynaptically located H1- or H2Rs were not essentially contributing in neuronal circuits involved in the ST-713-provided enhancing effects on behavioral deficits of tested BTBR mice.

MAPK/ERK signaling regulates many cellular processes by modulating the transcriptional and translational processes. Aberrant synthesis of synaptic proteins may contribute to ASD and ASD-like clinical features [56,57]. MAPK is responsible for synaptogenesis, and it regulates neural progenitor biogenesis, learning, and memory [58]. The increased levels of the MAPK pathway may be the cause of aberrant protein synthesis. Our observations showed that there were increased levels of ERK in vehicle-treated BTBR mice. The increased levels of ERK may have particularly increased the transcriptional process of adhesion molecules and scaffolding proteins, which caused imbalance in neuronal synapses, leading to the impaired cognitive functions and ASD-like features of examined mice [59,60]. Moreover, Faridar et al. 2014 suggested that hyperactivation of the CNS-associated ERK pathway may play an essential role in prefrontal-mediated social impairment [60]. Our observed results confirmed that a dose of 5 mg/kg of the test compound was able to prevent the increase in ERK levels, a finding that sheds light on the potential of ST-713 to normalize social behaviors of assessed animals.

The role of p38 in neuroinflammation is well reported, particularly its involvement in the synthesis of proinflammatory cytokines. Naturally, p38 is activated through many extracellular factors including cytokines, chemokines, and bacterial lipopolysaccharides; it regulates the cytokine expression that may be altering the nuclear transcription factor-kB (NF-kB) [61]. Our results suggest that treatment with ST-713 reduced p38-overexpression through the overtranslocation of p38 at the NF-kB site, and thereby decreased the levels of proinflammatory cytokines. These results agree with our previous observations that indicated that acute treatment with 5 mg/kg of ST-713 considerably decreased the expression of NF-kB in autistic mice [44].

In addition, JNK is reported to play a vital role in the microglial inflammatory response, and suppression of JNK activation with pharmacological inhibitors can also additionally reduce chronic inflammation and microglial activation [62]. During extra/intracellular stress in the brain, activated microglial cells can also cause white matter injury by induction of inflammatory cytokines [63]. As expected, 5 mg/kg of our brain-penetrant multiple-active test compound ST-713 markedly mitigated the protein expression of JNK, which can be the cause for the increased production of IL-10 and other proinflammatory cytokines [64]. In the autistic brain in previous preclinical studies, several proinflammatory cytokines, including TNF-α, IL-1β, and IL-6, were proven to be significantly increased [18,44,65,66,67]. Our latest observations confirmed these findings, showing that TNF-α, IL-1β, and IL-6 levels were considerably higher in the hippocampus and cerebellum of BTBR mice in comparison to age-matched control B6 animals. However, prolonged systemic pretreatment with ST-713 at all tested doses significantly reduced the elevated levels of proinflammatory cytokines in BTBR animals. Moreover, the reference drug chlorpromazine showed similar significant reducing effects on the assessed proinflammatory cytokines. In contrast, the levels of proinflammatory cytokines were increased when ST-713 was co-administered with the brain-penetrating H3R agonist (*R*)-α-methylhistamine, revealing that ST-713 provided beneficial effects (Table 1). The latter observation indicates that the effects observed by ST-713 were obtained through interactions with histamine H3Rs and that brain HA was involved in the mediated neuroprotective role of ST-713 on ASD-like symptoms of BTBR mice. Moreover, the observed results showed that the significant decrease in brain levels of HA and DA was moderately corrected following chronic systemic administration with ST-713 (Table 2), suggesting the capability of our test compound in its multiple-targeting approach for different neurotransmitters involved in the pathophysiology of neuropsychiatric disorders, e.g., ASD. Our results are in line with previous studies, as chlorpromazine has been found to have a slight antihistaminic activity, which may explain its failure to modulate HA levels and aggressive behaviors in tested mice. On the other hand and in addition to its D2/D3-antagonistic effect, ST-713 has an H3R-antagonistic effect, which may mediate the release of different brain neurotransmitters in several specific brain areas besides HA, such as DA, serotonin, and ACh, [28,68,69], and these effects are reflected in the observed modulation of HA and DA in hippocampus and cerebellum, leading to the witnessed improvement in the aggressive behavior in assessed BTBR mice. Recent reports from our group demonstrated a reduction in the elevated levels of COX-2, iNOS, and NF-kB in the brain through acute systemic administration with the same multiple-active test compound, namely ST-713 [44]. The current and previous findings for ST-713 in BTBR mice are significant, and they corroborate previous findings that deletion of brain HA in histidine-decarboxylase-knockout mice showed the importance of HA and histaminergic neurotransmission in maintaining the levels of the brain’s proinflammatory cytokines [70]. Moreover, previous preclinical studies have shown that individuals with neuropsychiatric disorders as well as ASD have chronic neuroinflammatory processes including microglial activation in several brain areas [71]. Consequently, this microglial activation may result in under-connectivity because of the continuous release of several mediators and the loss of synaptic connections, which in turn may cause the death of neuronal cells. This is crucial since several previous investigations have described under-connectivity in individuals diagnosed with ASD [72]. Our current findings showed considerably increased expression of iba-1-positive microglial cells in BTBR cerebellum compared with B6 mice, indicating an increase in microglial activation, and these findings are consistent with those of other studies reporting activation of microglial cells in BTBR brains. The latter observations for the significant increase in iba-1-positive microglial cells in BTBR mice brains reflect higher inflammatory processes through microglial activation in BTBR brains. However, chronic systemic treatment with ST-713 (5 mg) significantly lowered activated microglial cells in BTBR, as shown by the reduced expression of iba-1 proteins. In addition, the suppression displayed by ST-713 (5 mg) was reversed by concomitant administration of the H3R agonist (*R*)-α-methylhistamine. The latter findings suggested the capability of ST-713 to suppress activation of the microglial cells via an antagonistic interaction with H3Rs. As a result, the overall neuroprotection observed by ST-713 might be due to the enhancement of brain neurotransmitters such as HA, DA, and ACh, all of which are accountable for obvious ASD-like features witnessed in BTBR mice.

Notably, H3Rs have also been shown to form a heteromeric complex with D1R and D2R in cells and to co-immunoprecipitate with D1R or D2R in the striatum [73,74]. As H3R has been implicated in a number of psychiatric disorders, including schizophrenia, addiction, and ADHD [28,68,75,76], further future investigations for ST-713 with its multiple-targeting profile are warranted to comprehend whether the observed enhancing effects of ST-713 are due its capability of targeting the D1-, D2-, and/or -H3R heterodimers or solely the H3Rs.

## 4. Materials and Methods 

### 4.1. Animals

Male BTBR and C57BL/6J (referred to as B6) mice (The Jackson Laboratory, Bar Harbor, ME, USA) were used to conduct the behavioral experiments. All mice were 10–12 weeks old and weighed 28–32 g at the beginning of the study.. The animals were maintained at the CMHS animal facility, UAE University. The experimental animals were kept in an air-conditioned room which was isolated with controlled temperature and humidity (24 ± 2 °C and 55% ± 15%, respectively). All animals used in the experiments had free access to food and water. The experiments were conducted between 9.00 am and 3.00 pm. All experiments were approved (ERA-2019-6013) by the Institutional Animal Ethics Committee of the CMHS, UAE University. The smallest number of animals possible was employed in the current series of experiments to minimize the suffering of tested animals while maintaining the aim of the study.

### 4.2. Drugs and Reagents

The test compound was developed and profiled on its in vitro antagonist affinities in the Institute of Pharmaceutical and Medicinal Chemistry of Heinrich Heine University, Düsseldorf, Germany, following protocols described previously [31]. The multiple-targeting test compound ST-713 binds to H3R with high and selective binding affinities and balanced in vitro affinities to *h*D2/*h*D3R [31]. Chlorpromazine (CPZ, 1.5 mg/kg) was used as a reference drug. Scopolamine (SCO, 0.3 mg/kg), CNS-penetrant H1R pyrilamine (PYR, 10 mg/kg), H2R antagonist zolantidine (ZOL, 10 mg/kg), and centrally acting H3R agonist (*R*)-α-methylhistamine (RAM, 10 mg/kg) were procured from Sigma- Aldrich. All compounds were suspended in a 0.9% normal saline solution with a 1% aqueous Tween 80 solution. A VEH volume of 10 mL/kg i.p. injection was administered to each mouse adjusted to its body weight. The dosages of the test compound and the standard reference drug chlorpromazine are expressed in terms of their free bases. R&D Systems provided commercially accessible enzyme-linked immunosorbent assay (ELISA) kits for estimating the amount of proinflammatory cytokines (IL-1β, IL-6, and TNF-α) (Minneapolis, MN, USA). The dopamine (BioVision Catalog no: K4219-100) and histamine (Abcam Catalog no: ab213975) ELISA kits were used to evaluate the brain levels of histamine and dopamine following the instructions of the manufacturer.

### 4.3. Study Design

BTBR mice are an idiopathic model of ASD with abnormalities in their peripheral and central nervous system inflammatory profiles, and display characteristics similar to those found in people with ASD [1,71,77]. Moreover, this mouse strain shows dysregulated behavioral patterns, such as recurrent self-grooming behavior traits, deficiencies in social communication, and repetitive/compulsive activities that are comparable to those found in human ASD participants. Recently, and in our laboratories, ST-713 has shown promising ameliorative effects on social deficits of BTBR mice following its acute systemic administration [44].

All the animals in the study received chronic treatment (21 days). B6 mice functioned as the control group (group 1) and were injected with VEH. Mice with ASD-like behaviors treated with VEH (group 2) served as the control group of autistic-like mice. Varying dosages (2.5–10 mg/kg, i.p.) were administered in BTBR mice (groups 3–5). Chlorpromazine served as a reference compound (1.5 mg/kg) and was administered to BTBR mice (group 6). For the abrogation studies, 5 mg/kg of our multiple-active test compound was co-administered with pyrilamine (10 mg/kg, group 7), zolantidine (10 mg/kg, group 8), scopolamine (0.3 mg/kg, group 9), or (*R*)-α-methylhistamine (10 mg/kg, group 10) in BTBR mice. To exclude its confounding effects, BTBR mice were injected with (*R*)-α-methylhistamine (10 mg/kg, group 11). In addition to the 11 groups described above, 4 groups of six B6 mice each received ST-713 (2.5–10 mg/kg) as well as the reference medication chlorpromazine to exclude any confounding properties of the test compounds on the behaviors of control B6 mice. For the self-grooming assessment, a total of 15 mouse groups (6 mice/group) were used.

### 4.4. Behavioral Tests

After assessment of self-grooming behaviors was completed, different mouse groups were used to carry out the behaviors in the tube test. 

#### 4.4.1. Repetitive Self-Grooming Behaviors

As previously documented [65,66], test mice were evaluated for spontaneous grooming habits. Each mouse was housed in a conventional mouse cage independently (46 cm length × 23.5 cm wide × 20 cm high), illuminated at B 40 lux. To reduce neophobia, a thin (1 cm) layer of bedding was provided in each cage, while preventing digging as a possibly competing behavior. Prior to the test, mice were allowed 5 min of habituation to the test cage, then each mouse’s cumulative time spent grooming was measured using a timer for 10 min for each mouse. 

#### 4.4.2. Tube Test for Aggressive Behaviors

The tube test is a reliable behavioral task to evaluate cognitive functions in mice models of brain disorders, mainly social dominance, accomplished by the assessment of aggressive behaviors of tested rodents [78]. The tube dominance test was performed accordingly as described previously by Greco et al. [78]. In detail and before the test started, pre-training was given for three consecutive days to each mouse. The test tube was made up of clear Plexiglas material (30 cm long × 3 cm diameter), and mice were allowed to explore the tube by entering and passing through the tube. The test tube used in the current experiment was narrow enough (diameter of 3 cm) to avoid test mice being able to turn back or to pass each other during the test. In this test, the more social the test mice, the more social interaction will be present between the two tested animals, rather than trying to push out each other. During the pre-training, each mouse went from front to back of the tube three times (in total, six passes). The test consisted of two start areas, a two-section tube, and one neutral area between the two sections. To conduct the behavioral assessment, two age- and weight-matched mice were used (one B6 control mouse and one BTBR mouse with similar body weights since a large difference in body weight of the two assessed mice could influence the loss/win ratio). The mice were kept at either end of the tube, positioned headfirst, and released at the same time. Both gates were removed allowing the two mice to approach each other. The test ended when one retreated from the tube and was assigned a score of zero (lose); the remaining mouse was assigned a score of one (win). Mice that failed to achieve a win/loss outcome in 4 min were excluded from the test. The average value of three matches of each mouse was calculated and converted to average percentage of wins, then the mean percentage was calculated for each group. All 11 groups described above were used in this test, in addition to which 4 groups of six B6 mice each were chronically treated with ST-713 (2.5, 5, and 10 mg/kg) and chlorpromazine (CPZ 1.5 mg/kg) to exclude any effect of doses of test compounds on spontaneous locomotor activity of the tested groups. To evaluate the effects on aggressive behaviors in test tube assessments, 15 groups composed of 6 mice/group were used.

### 4.5. Biochemical Measurements

#### 4.5.1. Brain Collection and Tissue Processing for Proinflammatory Markers, Dopamine, and Histamine Level Analysis of Collected Mouse Brains

Following behavioral experiments, all the animals were sacrificed and analyzed for proinflammatory markers with ELISA according to our previously published procedures [43,44,79,80]. Briefly, pentobarbital (40 mg/kg body weight) was used as the anesthetic to allow the animals to go into deep sleep. Heart perfusion through transcardial infusion using 1× phosphate buffered saline was carried out and the brains were harvested and kept on an ice-cold plate for further dissections. For biochemical measurements, the cerebellum and hippocampus brain parts were separated and flash-frozen in a liquid nitrogen flask. Before starting biochemical assessments, all the tissue samples were weighed to an equal weight of 40 mg of hippocampus and 100 mg of cerebellum and then homogenized with ice-cold RIPA buffer. To avoid protein degradation, a protease and phosphatase inhibitor cocktail was added to the RIPA buffer; the homogenized samples were then centrifuged in a cooling centrifuge (4 °C) for 30 min at 12,000 rpm. The supernatant from each sample was collected for estimations of proinflammatory cytokines, dopamine, and histamine. From each group four animals were taken. After being transcardially perfused with PBS, the animals were further perfused and fixed with a 4% paraformaldehyde solution for immunofluorescence analysis. The brains collected were then postfixed in the same fixative (4% paraformaldehyde) for 48 h and successively exchanged with 10% sucrose solution for 3 consecutive days (4 °C). The brains were then stored at −80 °C for cryostat sectioning.

#### 4.5.2. Pro-Inflammatory Cytokine Estimations 

Quantification of the levels of pro-inflammatory cytokines (TNF-α, IL-1β, and IL-6) in the hippocampus and cerebellum was performed using ELISA as per the instructions of the manufacturer and our previously published experimental protocols [43,44,79,80]. 

#### 4.5.3. Estimation of Histamine and Dopamine Brain Levels

The brain levels of dopamine and histamine were determined using an ELISA kit according to the instructions of the manufacturer and following our previously described protocols [81]. 

#### 4.5.4. Western Blot Analysis

All the homogenized samples were analyzed for the levels of protein concentration and measured as previously described [44]. The separated proteins on 12% gel were transferred onto pre-activated (by 100% methanol) polyvinylidene fluoride membrane (PVDF, Millipore) for 1 h at 50 V. Then the membranes were blocked with 5% BSA for 1 h at 4 °C. The membranes were incubated with a mouse monoclonal anti-GAPDH (dilution 1:1000; Santa Cruz Biotechnology, Santa Cruz, CA, USA), mouse monoclonal anti-p38 (dilution 1:1000; Santa Cruz Biotechnology, Santa Cruz, CA, USA), mouse monoclonal anti-JNK antibody (dilution 1:1000; Santa Cruz Biotechnology, Santa Cruz, CA, USA), and rabbit polyclonal anti-p44/42 MAPK (Erk1/2, (dilution 1:1000; Cell signaling technology, MA, USA) overnight at 4 °C. The following day, the membranes were incubated with a HRP-conjugated respective secondary antibody for 1h at room temperature after adequate washing with TBST. The protein bands for the appropriate antibodies were visualized using SuperSignal West Pico PLUS (Thermo Scientific) Chemiluminescent Substrate following the manufacturer’s instructions. ImageJ software (NIH, USA) was used to quantify the band intensity.

#### 4.5.5. Iba-Immunofluroscence Staining 

The brains collected and stored previously were sliced into 20 µm thick coronal sections using a cryostat machine. The immunofluorescence staining was performed as described previously for assessing iba-1-positive microglia. In brief, the 20 µm brain sections were washed twice with PBS prior to blocking (incubation with 10% normal goat serum in PBS, 0.3% Triton-X 100 at room temperature for 1 h). The sections were washed and incubated with primary antibody (1:700) at 4 °C for 12–16 h. The next day, brain slices were adequately washed with PBS, prior to incubation with the appropriate fluorescent secondary antibody (Alexa 488 anti-rabbit, 1:1000) for 1 h at room temperature. The brain slices were washed with PBS before mounting with the fluorescent mounting media Vectashield^®^. Further, a fluorescent microscope EVOS FL (Thermo Fisher Scientific) was utilized to visualize and take the fluorescent pictures. Determination of activated microglia was performed by randomly equally selecting three different areas with the use of ImageJ software. The total corrected cellular fluorescence (TCCF) was calculated as TCCF = integrated density-(area of selected cell × mean fluorescence of the background readings).

## 5. Statistical Analyses

The sample distribution or skewness of the data acquired in behavioral evaluations was assessed for normality (−1.5 to +1.5 was considered normally distributed). Subsequent to the normality tests, the outcomes of the drug treatments were examined using two-way analysis of variance (ANOVA) with the dose of drug and animals (either BTBR or B6 mice) as between-subjects factors, followed by a Tukey’s test for post hoc comparisons in the case of a significant main effect. The significance of the data collected for protein expressions, cytokines, iba-1, HA, and DA estimates was determined using a one-way ANOVA followed by a post hoc Tukey’s multiple comparison test. Statistical significance was set as *p* < 0.05. All data were expressed as mean  ±  standard error of mean (SEM). 

## 6. Conclusions

The multiple-active test compound ST-713 was assessed for its mitigating effects on ASD-like repetitive self-grooming, aggressive behaviors, iba-1 expression, and pro-inflammatory cytokines. By virtue of its multiple-targeting strategy, the elevated levels of various proinflammatory cytokines were reduced following chronic systemic administration of ST-713. In addition, the disturbed levels of hippocampal as well as cerebellar neurotransmitters HA and DA were modulated by the same multiple-active test compound. However, more in vivo tests are warranted to determine whether multiple-targeting compounds, such as ST-713 (systemic treatment), are more beneficial than H3R antagonists/inverse agonists, such as pitolisant or ciproxifan, or antipsychotics, such as chlorpromazine, when used alone. In addition, further behavioral assessments using ST-713 are needed in different types of ASD-mutant mice, and by extension, in a range of other animal models with neurodevelopmental disorders, to generalize our current findings.

## Figures and Tables

**Table 1:** ST-713 mitigated levels of hippocampal and cerebellar proinflammatory cytokines in BTBR mice.

	Hippocampus			Cerebellum		
	*Proinflammatory cytokines*			*Proinflammatory cytokines*		
Treatment Group	TNF-α	IL-1β	IL-6	TNF-α	IL-1β	IL-6
B6 (Ctrl) (VEH)	96.98 ± 9.10	21.17 ± 2.41	49.92 ± 9.68	303.15 ± 7.20	241.69 ± 11.85	48.29 ± 6.03
BTBR (Ctrl) (VEH)	205.87 ± 17.83 *	161.21 ± 12.18 *	181.45 ± 14.07 *	413.47 ± 12.89 *	385.63 ± 31.18 *	202.73 ± 10.74 *
BTBR (ST-713, 2.5 mg/kg)	116.45 ± 15.35 ^##^	98.36 ± 2.90 ^##^	69.36 ± 7.08 ^##^	253.37 ± 3.36 ^##^	182.64 ± 14.84 ^##^	92.33 ± 13.81 ^##^
BTBR (ST-713, 5 mg/kg)	105.26 ± 10.08 ^##^	81.35 ± 8.26 ^##^	70.32 ± 14.63 ^##^	263.90 ± 5.52 ^##^	205.85 ± 16.74 ^##^	82.54 ± 4.95 ^##^
BTBR (ST-713, 10 mg/kg)	119.20 ± 10.42 ^##^	82.90 ± 9.72 ^##^	67.95 ± 13.17 ^##^	268.67 ± 5.25 ^##^	229.50 ± 19.14 ^##^	119.30 ± 10.92 ^##^
BTBR (CPZ, 1.5 mg/kg)	47.05 ± 4.65 ^##^	27.61 ± 4.19 ^##^	56.14 ± 3.60 ^##^	267.70 ± 8.15 ^##^	233.65 ± 21.62 ^##^	51.04 ± 9.53 ^##^
BTBR (ST-713, 5 mg)+ RAM	178.88 ± 22.09 ^$^	137.30 ± 4.71 ^$^	150.04 ± 10.24 ^$^	336.04 ± 16.57 ^$^	378.16 ± 25.34 ^$^	142.12 ± 7.95 ^$^

Modulated Tumor Necrosis Factor (TNF-α, pg/mg protein), interleukin-1β (IL-1β, pg/mg protein), and interleukin-6 (IL-6, pg/mg protein) were assessed. BTBR mice showed a significant increase in TNF-α, IL-1β, and IL-6 in the hippocampus and cerebellum compared to B6 mice. Test compound (2.5–10 mg/kg) or chlorpromazine (1.5 mg/kg) were administered chronically for 21 days in BTBR mice. Test compound or chlorpromazine significantly decreased TNF-α, IL-1β, and IL-6. Effects of chronic (21 days) systemic co-injection of RAM (10 mg/kg) on test-compound (5 mg)-mediated alteration of the levels of proinflammatory cytokines were evaluated. Data are expressed as the mean ± SEM (n = 6). * *p* < 0.05 vs. VEH-treated B6 mice. ^##^ *p* < 0.01 vs. BTBR mice. ^$^ *p* < 0.05 vs. ST-713(5 mg)-treated BTBR mice. ND; not determined.

**Table 2:** ST-713 modulated levels of hippocampal and cerebellar HA and DA in BTBR mice.

	Hippocampus		Cerebellum	
Treatment Group	HA	DA	HA	DA
B6 (Ctrl) (VEH)	0.49 ± 0.04	54.87 ± 0.87	0.48 ± 0.04	43.47 ± 3.62
BTBR (Ctrl) (VEH)	0.37 ± 0.03 *	47.75 ± 2.19 **	0.37 ± 0.00 *	42.05 ± 0.64
BTBR (ST-713, 2.5 mg/kg)	ND	ND	ND	ND
BTBR (ST-713, 5 mg/kg)	0.78 ± 0.06 ^###^	54.48 ± 0.52 ^#^	0.47 ± 0.02 ^###^	42.55 ± 1.33
BTBR (ST-713, 10 mg/kg)	ND	ND	ND	ND
BTBR (CPZ, 1.5 mg/kg)	0.41 ± 0.06	46.56 ± 1.44	0.39 ± 0.01	42.64 ± 3.02
BTBR (ST-713, 5 mg) + RAM	0.40 ± 0.02 ^$^	46.58 ± 2.23 ^$^	0.34 ± 0.01 ^$^	42.34 ± 0.53

Modulated brain levels of histamine (HA, ng/mg protein) and dopamine (DA, ng/mg protein) were assessed. BTBR mice showed a significant decrease in HA and DA in hippocampus and cerebellum compared to B6 mice. Test compound (2.5–10 mg/kg) or chlorpromazine (1.5 mg/kg) were administered chronically for 21 days in BTBR mice. ST-713 at 5 mg/kg considerably modulated disturbed brain levels of histamine (HA) and dopamine (DA). Effects of chronic (21 days) systemic co-injection of RAM (10 mg/kg) on test-compound (5 mg)-mediated alteration of the levels of HA and DA were evaluated. Data are expressed as the mean ± SEM (n = 6). * *p* < 0.05 vs. VEH-treated B6 mice. ** *p* < 0.01 vs. VEH-treated B6 mice. ^#^ *p* < 0.05 vs. BTBR mice. ^###^ *p* < 0.001 vs. VEH-treated BTBR mice. ^$^ *p* < 0.05 vs. ST-713(5 mg)-treated BTBR mice. ND; not determined.

## Data Availability

Data are contained within the article.
